# PROTOCOL: Interactive social media interventions for health behaviour change, health outcomes, and health equity in the adult population

**DOI:** 10.1002/CL2.213

**Published:** 2018-04-05

**Authors:** Vivian Welch, Jennifer Petkovic, Rosiane Simeon, Justin Presseau, Diane Gagnon, Alomgir Hossain, Jordi Pardo Pardo, Kevin Pottie, Tamara Rader, Alexandra Sokolovski, Manosila Yoganathan, Peter Tugwell, Marie DesMeules

## BACKGROUND

### The problem

With three billion Internet users globally (ITU 2014), more than two billion of whom are estimated to be active social media users (We Are Social 2016), social networking platforms such as Facebook and Twitter present an opportunity to reach large numbers of Internet users quickly with health information. For public health authorities, health promotion agencies, non‐governmental organisations, and others, social media offers an especially attractive opportunity to communicate with target audiences because these tools are generally easy and free to use and may allow organisations to reach a broad population, providing they have Internet access, including rural and remote populations. Furthermore, social media allows target audiences for health‐related interventions to share information and comments on topics that are of interest to them. In this way, organisations with relevant and informative health‐related campaigns may reach broader audiences through the social networks of users who follow them.

**Figure 1:**
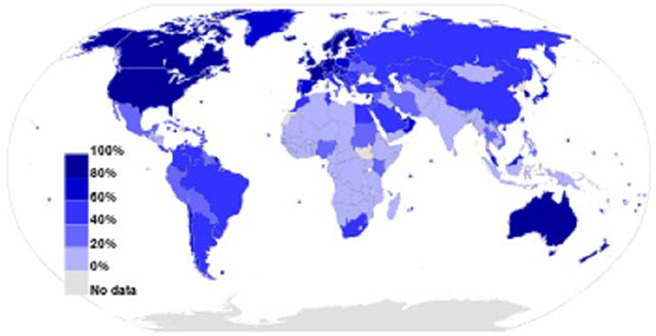
Internet users 2012 as a percentage of a country's population

**Figure 2:**
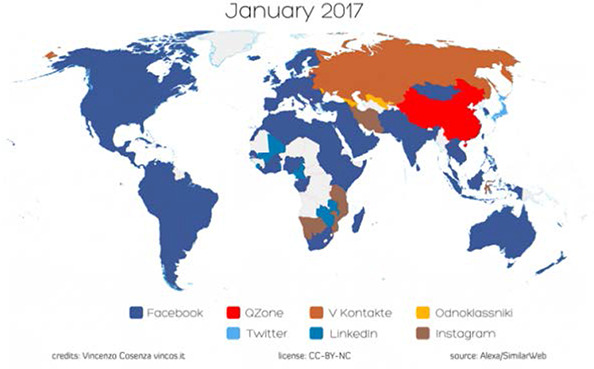
World Map of Social Media

It is important to note that Internet access and usage vary within and between countries and world regions, as evidenced by the fact that the International Telecommunication Union (ITU) estimates that, as of 2016, Internet users range from a high of 79% in Europe to a low of 25% in Africa (ITU 2016). In 2012, 83% of Canadians aged 16 or over used the Internet for personal use, and 67% of those Internet users visited social networking sites such as Facebook or Twitter (Statistics Canada 2013). What is more, as of 2012 almost 70% of Canadian Internet users searched online for medical or health‐related information (Statistics Canada 2013). Similar rates have been seen in the United States, where 72% of adult Internet users report that they have searched online for information about a variety of health issues, the most popular being specific diseases and treatments (Fox 2014). (See Figure 1). In countries such as Canada and the United States, income has been shown to be a key source of digital inequality and is not only a significant determinant of Internet access, but also online activity level (Haight 2014). Ninety‐five per cent of Canadians in the highest income quartile have Internet access, whereas only 62% of those in the lowest income quartile are connected (Canadian Internet Registration Authority 2014). There is a risk that people who experience health inequity may face barriers to the use of social media, such as access, reading literacy and/or electronic health literacy (NCCHPC 2015; Welch 2016). Social media use is lower in low‐ and middle‐income countries. A 2014 survey of 32 emerging and developing nations found that those who read or speak English are more likely to access the Internet and Internet access and smartphone ownership rates were found to be greatest among the well‐educated and 18‐ to 34‐year‐olds (Pew Research Center 2015). Thus, social media interventions may inadvertently exacerbate health inequities if those who are most disadvantaged are excluded from participation due to these issues. (See Figure 2).

Health equity is a major focus of both policy and research organisations from the local to the global level, such as the World Health Organization. Health inequities are differences in health that are avoidable and unfair (Whitehead 2006). For the purpose of this review, we use the PROGRESS‐Plus framework to consider socially stratifying characteristics that are associated with inequities in health (O'Neill 2014; Welch 2016). Coined by Evans to describe characteristics that may contribute to health inequity, PROGRESS stands for Place of residence, Race/ethnicity/culture/language, Occupation, Gender/sex, Religion, Education, Socioeconomic status, and Social capital (Evans 2003). ‘Plus’ represents personal characteristics that are associated with discrimination (e.g. age, disability), features of a relationship (e.g. smoking parents, excluded from school), time‐dependant relationships (e.g. leaving the hospital, respite care) and other circumstances that may be related to health inequities (Gough 2012).

Social networking sites popular at the time of this review, like Facebook, Twitter, YouTube, and LinkedIn, as well as related apps, are designed to promote the sharing of information and opinions in the form of text, images, and video among friends, family, acquaintances, and associates as well as public figures, businesses, and other organisations with whom users associate by ‘following’ and ‘liking’ pages or accounts. Health‐specific social media have piggybacked on the most popular features of social networking sites in order to provide support for people who share an interest in a particular health concern, such as depression or weight loss. Furthermore, there is evidence that social media use may create a sense of community among people, giving users a feeling of being supported and accepted (Dyson 2015). As noted by Vitak 2014, numerous researchers have found a positive correlation between social media use and social capital, “a construct that encompasses both actual and potential resources available within a given network”.

However, with the speed and reach of social media‐based communication come attendant risks that may compromise health, such as the potential for equally rapid diffusion of misinformation or information that is not scientifically based. For example, the anti‐vaccination Facebook page vactruth.com is ‘liked’ by almost 100,000 Facebook users and some of its posts are shared hundreds of times, meaning that its content may be seen by many more people than the number of page followers suggests. What is more, social media is sometimes used as part of a wider campaign to change cultural norms and behaviours, sometimes in harmful ways, such as the use of social media as part of campaigns by the alcohol industry in countries such as Australia (Westberg 2016). In addition, the use of social media itself may be associated with adverse outcomes unrelated to the actual intervention or social media platform. These include perceived social isolation, depression and anxiety, cyberbullying, sexting, or privacy concerns (O'Keeffe 2011; Tromholt 2016; Primack 2017).

As opposed to interventions where changes are introduced without the direct involvement of affected populations, such as encouraging food manufacturers to reduce sodium content in their products, social media interventions require a high level of ‘agency’, or personal resources and effort on the part of participants (Adams 2016). Therefore, there is the potential for intervention‐generated inequities because social media interventions require the use of personal resources such as time, material resources or cognitive resources, all of which may be less available for people experiencing health inequities due to competing demands on time and resources (Lorenc 2013; Adams 2016). Combining social media with other ‘low agency’ interventions such as policies to reduce the salt content of food may have a synergistic action by promoting acceptability of ‘low agency’ interventions, which can be seen as paternalistic and controlling. Thus, the combination of social media with other initiatives, such as population level changes in food policies or environment, may have synergistic effects.

### The intervention

For the purpose of this review, social media is defined as “activities, practices, and behaviours among communities of people who gather online to share information, knowledge, and opinions using conversational media…that make it possible to create and easily transmit content in the form of words, pictures, videos, and audios” (Safko 2012). Health‐related social media interventions for adults use social networking sites to promote a message that may influence health service uptake, health behaviour change (such as smoking, physical activity, or diet), and, depending on the nature of the intervention, health outcomes such as weight loss, depression, or quality of life.

Young 2015 provides an example of a social media‐based intervention aiming to improve a health behaviour among vulnerable groups – in this case, uptake of free HIV testing among men who have sex with men. This randomised trial was developed by researchers at the University of California, Los Angeles in partnership with a local community clinic in Lima, Peru to test the efficacy of peer mentorship offered through Facebook. Investigators created non‐public Facebook groups, which were joined by members of both the intervention and control groups. However, intervention groups included trained peer leaders who attempted to discuss with other members the importance of HIV prevention and testing, whereas the online community in which control group participants joined had no peer leaders, and participants simply received HIV testing information. Thus, intervention groups were subject to a more intensive social media intervention than control groups. These social media interventions represented an enhancement of standard of care provided by local community clinics and government organisations in Lima, which entails providing HIV prevention and testing services for public use.

Another example of a social media intervention is described by Maher (Maher 2015). A free, 50‐day team‐based Facebook app called Active Team was developed by a team at the University of South Australia. As part of a randomised controlled trial, insufficiently active adult participants were recruited and allocated to either the intervention group or the control group. In the intervention group, participants were given a pedometer and encouraged to take 10,000 steps per day as part of a team of three to eight existing Facebook friends who the app encourages to engage in friendly rivalry and peer encouragement and support. The app includes a calendar for logging daily step counts, a dashboard showing step‐logging progress, awards, and gifts, and a team tally board so that users can monitor personal progress and their friends' progress. It also includes a team message board where team members can communicate, daily tips for increasing physical activity, and other features intended to be fun and encouraging to use. Control group members were wait‐listed for the app‐based intervention and followed up with the same measurements as the intervention group.

### How the intervention might work

For this review, we will focus on ‘interactive social media’ in which the intervention allows for two‐way communication between peers or the public. This interactive functionality of social media offers a tremendous opportunity for increasing the reach of health interventions and enhancing a person's ability to engage in healthful behaviours. In addition to its potential to facilitate interactions between institutional providers and populations, social media allows lay people to create health‐focused groups to communicate with peers (Ali 2015; Myneni 2016). Furthermore, widespread use of mobile phones and other smart devices coupled with access to high‐speed Internet have considerably increased the ubiquitous functionality of social media while undermining limitations related to geographical locations, times, and social and economic status (Uskov 2015). In addition, because of the penetration of social media globally, people have experience using these interfaces that may allow them to take advantage of their functionality for finding, sharing, and using information.

Interactive social media has the potential to uphold health endeavours in various ways. Recent studies have reported the use of social media in strategies aimed at influencing individual health behaviours, informing health research, supporting health advocacy groups, and promoting health services (Brusse 2014; Seltzer 2015; Rhodes 2016; Sinnenberg 2016; Wong 2016). While the use of social media is common for supporting public health activities, very few organisations have reported consistent strategies describing how public health interventions sustained through social media have helped achieve their health equity goals (Thackeray 2012; Osborne 2013; Chauvin 2016; Ndumbe‐Eyoh 2016).

**Figure 3:**
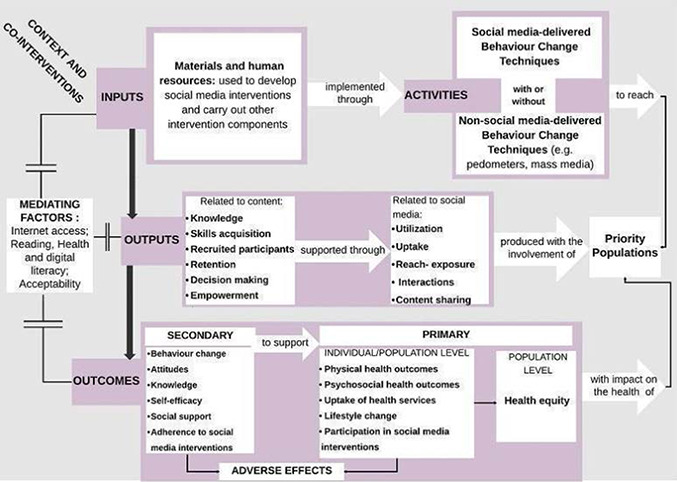
Logic model describing social media interventions for improving health and health equity

**Figure 4:**
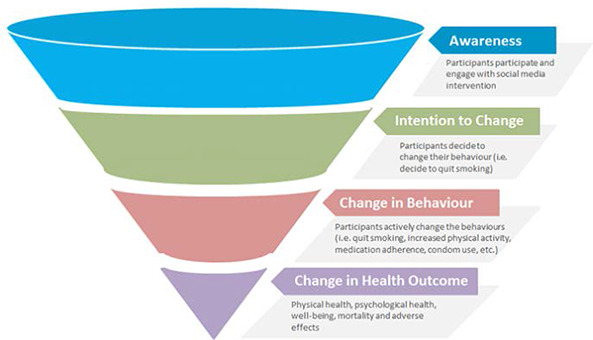
Funnel of Attrition

The logic model, developed by our team for this review, displayed in Figure 3 illustrates the components of social media interventions, including resources and behaviour change techniques (BCTs) (both within social media platforms as well as outside of social media), their expected intermediate (secondary) outcomes (e.g. on knowledge acquisition, attitudes, self‐efficacy, motivation, emotions, and ultimately behaviour change), outcomes (e.g. physical and psychological health, health equity), and potential for adverse effects. We will use the logic model to explore effects along the causal chain, and consider adapting the logic model with evidence from the review (if needed). We aapted the Funnel of Attrition to describe the mechanism of action of social media interventions on outcomes of interest (see Figure 4) (Waddington 2014).

One of the reasons for using social media to deliver public health interventions is its capacity to build and reinforce social support for improving health outcomes (Osborne 2013; Vassilev 2014; Rhodes 2016; Rice 2016). Enabling social support through interactive social media has been linked to positive impacts on health outcomes by influencing knowledge, motivation, self‐efficacy (one's perceived ability to perform a behaviour), and other beliefs and cognitions towards health behaviours (Bandura 2000; Rhodes 2016; Rice 2016; Wong 2016). When used as a means for strengthening social networks, interactive social media may help promote public health and health equity by fostering collective efficacy (Greene 2011; Phua 2013; Di Bitonto 2015). Collective efficacy, a construct of social cognitive theory, is defined as “people's shared beliefs in their collective power to produce desired results” (Bandura 2000). Additional to the structure and the goal of the group, achieving group efficacy may be conditioned by self‐efficacy, social comparison, or other specific rules governing the overall functioning of the group as one unit (Bandura 2000; MacAlister 2008; Ross 2010; Zhang 2016). Given the difficulty of anticipating the structure of participating communities in social media interventions and the dynamic underpinning the functioning of online groups, collective efficacy was not included in the proposed logic model, however, we will collect information on collective efficacy, if it is reported in studies.

Social media is often used in public health as a platform or setting for sharing knowledge, building skills, expanding the reach of public health interventions, fostering empowerment, and facilitating decision‐making among priority populations (Seltzer 2015; Hudnut‐Beumler 2016; Ndumbe‐Eyoh 2016). The logic model (Figure 3) acknowledges that interventions that involve social media are often complex interventions (Craig 2008), involving multiple components including offline intervention components to reinforce the message of the health‐related campaign.

Nevertheless, varied levels of interest in, access to, and acceptance of e‐technologies have been reported as affecting the uptake and effectiveness of social media interventions for public health (Thackeray 2012; Antheunis 2013; Merolli 2013; Uskov 2015). Other studies have highlighted the mediating effect of characteristics such as social position, familiarity with social media, and literacy (reading, health, and digital literacy) in boosting the effect of social media interventions on health outcomes (Korda 2013; Merolli 2013; Osborne 2013; Real 2015; Rice 2016). These elements have relevance for the replication of the interventions studied and should be factored into any description of the effects of social media interventions on health outcomes.

Other concurrent public health initiatives, such as campaigns or community mobilisation, and the context in which social media interventions are implemented may also impact the effectiveness of such interventions towards achieving health equity (Hudnut‐Beumler 2016; Ndumbe‐Eyoh 2016; Rice 2016). Thus, the effects of social media combined with campaigns should be interpreted with caution and their adaptation should be based on a thorough analysis of the needs of priority populations and assets available within the communities of interest.

Adverse and unintended effects from communication campaigns may arise due to stigmatisation and other reasons. For example, stigmatisation may be reported in interventions that use mechanisms such as self‐presentation and social comparison to promote healthy behaviours. Self‐presentation is described as “behavior that attempts to convey some information or image of oneself to other people” and it is often motivated by situational factors (Baumeister 1987). On the other hand, social comparison consists of drawing on others' behaviours to make comparison with one's performance (White 2006). Self‐presentation and social comparison have been reported as beneficial in interventions delivered online aimed at promoting healthy behaviours in the context of HIV prevention and physical activity, respectively (Byron 2013; Zhang 2016). While serving positive functions such as avoiding harms and encouraging healthy behaviours, self‐presentation and social comparison may also generate stigmatisation and embarrassment when strategies like manipulation and exemplification are used (Baumeister 1987; White 2006; Byron 2013). For example, someone may present themselves in such a way that they appear competent, dangerous, or morally virtuous (Baumeister 1987). Other adverse or unintended effects of communication campaigns can include confusion and misunderstanding about health risks and risk prevention and ‘boomerang’ (the reaction of the audience is the opposite of what was intended) (Cho 2007).

Fear of the consequences of privacy breaches and exposure of one's vulnerability through participating in the intervention may deter some individuals from enrolling. In order to avoid unwanted behaviours and to preserve the reputation of the interventions, organisers may establish consent processes that contain warnings to remove anybody deemed behaving inappropriately from the group. This situation may have the perverse effect of further excluding individuals who may have otherwise benefited from the public health intervention if the latter was not delivered through social media. Based on concerns over data security on social networking sites and researchers' experience with an HIV education intervention delivered through social media, it has been suggested that health researchers familiarise themselves with current privacy settings available in order to help protect participants, and that they educate participants on how to better safeguard their privacy (Bull 2012). Thus, privacy breaches are one potential adverse effect that may be of special interest for the purpose of health interventions delivered online, especially for sensitive topics like sexual practices.

Lack of understanding of the research process and informed consent on the part of participants may influence participation in social media interventions and may differ for specific population groups (e.g. low literacy), especially in studies where this information was provided to participants online rather than with the direct involvement of research study personnel. For example, in the Harnessing Online Peer Education randomised trial (Young 2015), participants received information about the study and completed informed consent online. Chiu 2016 found that younger HOPE study participants, who generally had less experience with research studies than older participants, were less likely to indicate that they had understood the consent form and study process.

### Why it is important to do the review

Although there are other systematic reviews of social media interventions, these reviews have concluded that there is a gap in knowledge on the effects of modern social media (Maher 2014; Merolli 2013), and their narrower scope limited their ability to explore the mechanisms of action and possible effect modifiers across different type of behaviours (Laranjo 2015; Williams 2014). Heffernan 2017 have a forthcoming Cochrane title on whether social media influences attitudes and uptake of vaccines; however, this review will focus solely on vaccination, which has a set of issues that may not be generalisable to other areas of public health. Hamm 2014 reviewed the use and effectiveness of social media in child health. Thus, we are focusing on adult social media users.

There are several Cochrane and non‐Cochrane systematic reviews on the effects of mass media interventions on topics as diverse as alcohol consumption, smoking prevention and cessation, HIV testing, mental health stigma, uptake of health services, and preventing non‐communicable diseases (Bala 2008; Brinn 2010; Clement 2013; Grilli 2002; Mosdøl 2017; Siegfried 2014; Vidanapathirana 2005). Our review differs from these because we are focusing on interactive social media interventions that allow exchange of ideas, not mass media.

A number of reviews have examined equity impacts of health interventions, including those relating to physical activity (Humphreys 2013), prevention, management, or reduction of obesity (Bambra 2015), under‐nutrition (Kristjansson 2015), and healthy eating (McGill 2015). However, the effects of social media interventions on disadvantaged populations have not been assessed in previous reviews. On one hand, social media interventions have the potential to reach geographically dispersed populations, whereas on the other, there may be barriers such as the digital divide, language, literacy, acceptability, and risk of intervention‐generated inequities. Thus, it is important to assess the effects of social media interventions on the health of disadvantaged populations.

## OBJECTIVES

### Primary objectives

To assess the effects on adults of interactive social media interventions on:

- health‐related behaviours;
- physical health outcomes;
- attitudes;
- any reported adverse effects

### Secondary objectives

- To assess the effects of interactive social media interventions that aim to change health behaviour across population subgroups (defined using PROGRESS‐Plus) to assess effects on health equity.
- To use a validated taxonomy of behaviour change techniques to determine whether social media interventions with specific behaviour change techniques (BCTs) (or BCT combinations) are more effective.
- To explore heterogeneity of effects to identify other reasons for differences in effects.

## METHODOLOGY

### Characteristics of studies relevant to the objectives of the review

### Criteria for inclusion and exclusion of studies in the review

#### Participants

A recently conducted systematic review examined the use and effectiveness of social media in child health (Hamm 2014), therefore our participants of interest include members of the general population who are 18 years of age and older. We will include studies with mixed populations (e.g. youth aged 15 to 24), if we can obtain disaggregated data for participants aged 18 years and older, or if the study reports that the population is mostly over 18 years of age (i.e. 70% or more of the population are 18 years of age or older). We will include people from the general population, including participants with an identified health condition.

Given that we are also interested in the effect of social media interventions on health equity, we will include studies that focus on or present disaggregated data across ‘PROGRESS‐Plus’ characteristics. We will also contact authors for more detail on the social media platforms (since we expect that these will not be fully described in the published articles) and to request whether the authors conducted analysis across PROGRESS‐Plus characteristics and, if so, whether they can share these data.

#### Interventions

We will use the Safko definition of social media: “activities, practices, and behaviours among communities of people who gather online to share information, knowledge, and opinions using conversational media…that make it possible to create and easily transmit content in the form of words, pictures, videos, and audios” (Safko 2012).

To be included in our review, the social media intervention must allow for interaction including two‐way communication between the user and peers. We will exclude any intervention that only offers one‐way communication as well as those that only offer one‐to‐one communication.

In addition, we will restrict inclusion to studies that focus on changing one or more behaviours. We will assess this using the following criteria:

- the study purpose is focused on changing one or more behaviours (e.g. exercise, smoking cessation); **or**
- the website/app or platform of the intervention tool describes a goal of changing behaviour; **or**
- the components of the intervention include a behaviour change technique documented in the Behaviour Change Technique taxonomy (Michie 2013; Presseau 2015).

We will only include social media interventions using commonly used social media tools (e.g. Facebook, Twitter) or those mimicking their interface (e.g. Quitnet) and related applications (apps). We will exclude web‐based chat rooms designed by researchers or others since these are no longer used, they do not have a user interface like these other commonly used tools, and they have been synthesised in our overview (Welch 2016). Furthermore, because these web‐based chat rooms are not familiar to users, they require a learning curve and an extra effort to engage with them that is not required by tools such as Facebook or Twitter, with which users have familiarity. Examples of the types of social media interventions to be included in this review are summarised in Table 1 (adapted from Welch 2016). We will include peer‐initiated interventions as well as interventions initiated by organisations such as public health organisations or private organisations (e.g. Weight Watchers).

**Table 1:** Types of social media interventions

**Social media format**	**Included**	**Excluded**
Blogs and microblogs (e.g. Twitter)	If the intervention includes multi‐way interaction between users (e.g. Twitter that promotes discussion)	Blogs would almost always be excluded since they usually have limited interaction. One‐way messages and posts or direct contact with a health care provider.
Content communities (e.g. YouTube, Pinterest)	If the intervention includes multi‐way interaction	One‐way messages and posts or direct contact with a health care provider
Mobile applications (apps)	Apps that allow for communication and interaction with a group of people	Apps that allow a person to track and monitor their progress (e.g. weight loss, blood sugar, etc.) without a social component or apps used to communicate with a health care provider
Virtual social networks (e.g. Facebook, Odnoklassniki)	If the intervention includes multi‐way interaction	One‐way messages and posts or direct contact with a health care provider
Web pages and Wikis	If the website/Wiki allows for multi‐way interaction	One‐way communication (e.g. education)

We will exclude studies of ‘beta’ interfaces that are aimed at assessing usability and improving the interface. These studies have limited applicability to understanding how social media can be used to influence health.

We will exclude studies assessing e‐health or telemedicine interventions that use technology to deliver health care. We will exclude studies that assess mobile health (e.g. apps that track clinical information with communication between an individual and their health care provider) and content that is transmitted unidirectionally (e.g. text message reminder interventions in which the recipient is unable to reply, podcasts in which health information is provided with no opportunity for two‐way communication) or which only allows for comments without sharing functionality, such as blogs. We will also exclude studies that assess online interventions that are based on exchange between a single care provider and a single participant such as online cognitive behavioural therapy, as they are covered within other reviews as telemedicine or e‐health interventions. Advertisements on social media (e.g. on Facebook) will be ineligible if they do not have sharing functionality. We will also exclude studies of virtual gaming interventions.

We will include studies comparing interactive social media interventions to usual care, no intervention, or an active comparison (e.g. one type of social media compared to another).

#### Outcomes

We will not exclude studies on the basis of outcome. However, studies with none of the primary or secondary outcomes will not be included in meta‐analysis.

#### Primary outcomes

The primary outcomes are physical health, psychological health, health behaviours (including accessing or using health services), well‐being, mortality, and adverse effects (e.g. stigmatisation, exclusion, or harmful health behaviours).

- Physical outcomes include any measures of physiological health such as body mass index (BMI), physical fitness, lung function, or asthma episodes, using validated measurement tools. Surrogate biochemical markers of physiological health such as haemoglobin A1C and viral load will not be included.
- Psychological health includes measures of depression, stress, coping, and other measures, using validated tools.
- Health behaviours include alcohol consumption, blood/organ donation, breastfeeding, dietary changes, levels of physical activity, medication adherence, illicit drug use, sexual behaviours, smoking, sun protection, and seeking and using health services, using validated measurement tools.
- Well‐being includes measures of quality of life, using any validated measurement tools.
- For adverse effects, we will document and report on any reported adverse outcomes or unintended consequences associated with social media interventions, such as online harassment and privacy concerns related to discussing or otherwise revealing health issues or health status online, and ethical issues pertaining to participants' privacy.

These types of outcomes are broad since we anticipate that studies that aim to change different behaviours will measure different types of behaviours and different types of physical and psychological outcomes. For example, a study that aims to increase exercise behaviours might measure body mass index, whereas a study that aims to reduce smoking might measure respiratory‐related morbidity. Thus, we will classify outcomes according to the above five types: physical health outcomes, psychological health outcomes, health behaviours, well‐being, and adverse effects. Within each type, we will include only outcomes measured with a validated measurement tool, and we will collect details of the validation of these measurement tools. We expect considerable heterogeneity in methods of measurement (e.g. self‐reported, computer‐collected) as well as measurement tools. In consultation with our clinical and statistical experts, we will assess whether it is appropriate to combine these different outcomes, based on conceptual similarity, using standardised mean differences, as described in the analysis section below. We expect that the mechanism of intervention will be similar for these different types of outcomes (see Figure 4) and we are interested in whether the social media interventions have an effect on these different outcome categories with less emphasis on the specific outcome measures. For example, we are interested in whether social media interventions that aim to change behaviours will result in changes to physical health outcomes and are less concerned about whether this is a change in waist circumference, BMI, or lung function. Therefore, we plan to pool outcomes according to these pre‐defined categories unless deemed inappropriate by our content and statistical experts.

When a study includes more than one measurement of our outcome classification above, we will seek to assess which outcome was considered primary in the trial, based on whether it was named as a primary outcome, used in a sample size calculation or reported more prominently in the abstract or results. We recognise that this may not be possible since some studies have multiple measures of the same concept (e.g. we have identified over 15 measures of exercise behaviour modification such as frequency, intensity, and type of activity, and some studies report three or more measures). Therefore, we will document how these decisions are made and what additional outcomes are available in the description of studies.

#### Secondary outcomes

To assess potential impact on health equity, we will collect and report data on population‐specific effects across PROGRESS‐Plus characteristics, if available. These data may be from studies focused on disadvantaged populations or they may be from studies aimed at a broader population, where subgroup analysis has been conducted to assess variations in effects across one or more PROGRESS‐Plus characteristic.

- Social media use/participation/adherence.
- Attitudes.
- Knowledge.
- Motivation and self‐efficacy.
- Other theory‐based constructs related to behaviour change (e.g. perceived social support)

We will also assess details of collective efficacy (using any method of measurement). As with the primary outcomes, we expect heterogeneity in how these outcomes are measured. We do not think it is possible to insist on a common measurement tool or method. However, we will only include validated measures of these concepts. We will classify all outcomes according to these constructs, then decide in consultation with our clinical and statistical experts whether it is possible to statistically combine these.

#### Research methods/designs

Based on our earlier overview of reviews (Welch 2016), and more recent reviews, we anticipate finding over 40 randomised trials of social media interventions. Since some types of social media, such as peer initiated social media, are not conducive to randomisation, we decided to limit this review to Cochrane Effective Practice and Organisation of Care recommended study designs (EPOC 2017a), as follows:

- Randomised controlled trials (RCTs): These studies consist of randomly assigning participants to receive one of the interventions studied. Participants may be assigned to interventions individually or by group (cluster‐RCTs). The interventions are usually described as treatment group (individuals who receive the intervention) and control group (individuals who do not receive the intervention.
- Controlled before‐and‐after (CBA): These studies consist of measuring outcomes before and after the implementation of an intervention in both the treatment group and control group. Study investigators are not involved in the assignment of participants to either treatment or control group. Allocation is usually determined by other factors outside the control of the investigators.
- Interrupted time series (ITS): These studies consist of measuring outcomes at multiple time points before and after an intervention (‘the interruption’) with the intent to capture whether the trends persist or there is a change in the outcomes measured after the intervention. When outcomes are assessed at regular intervals in the same participants, the ITS is called a repeated measures study (RMS).

We will accept RCTs with stepped‐wedge designs (treatments begun at different times for different groups of participants). In these cases, our baseline will be the time at which the ‘treated group’ (longest treatment) began treatment and our endpoint will be the point at which the ‘control group’ began treatment. We will exclude all other study types.

**Search strategy for finding eligible studies**

We will search for studies published between 2001 and the date of the search, since most of the commonly used social media platforms were developed in 2001 or later (e.g. Facebook, Twitter) and our overview showed no earlier studies using these commonly used social media applications (Welch 2016).

We will not include a language limit on the searches. Our team is able to collect data from studies in English, Spanish, Catalan, and French. We will seek help using Cochrane Task Exchange for studies in other languages.

#### Electronic searches

We searched the following bibliographic databases for eligible empirical studies.

- Cochrane Central Register of Controlled Trials (CENTRAL) (2001 to search date).
- MEDLINE and Pre‐MEDLINE (2001 to search date).
- EMBASE and EMBASE Classic (2001 to search date).
- Cumulative Index to Nursing and Allied Health Literature (CINAHL) (2001 to search date).
- PsycINFO (2001 to search date).

#### Searching other resources

We will also search for unpublished studies or reports that meet our eligibility criteria using a focused search within Google and Web of Science, as well as searching websites of public health governmental and non‐governmental organisations, such as the Public Health Agency of Canada, the World Health Organization (WHO), and international development agencies such as the Asian Development Bank and the Inter‐American Development Bank. We will also search clinical trials registries (ClinicalTrials.gov and the WHO International Clinical Trials Registry Platform (ICTRP) for relevant studies.

We will also contact authors of included studies to ask for suggested studies and scan the reference lists of included studies.

### Data extraction and study coding procedures

#### Selection of studies

Two review authors will independently screen titles and abstracts to identify relevant studies meeting the pre‐specified inclusion criteria using Covidence. We will screen in full text studies included at the title/abstract level. We will discuss and resolve disagreements by consensus or with a third member of the research team (VW) when necessary. We will extract data from all included studies.

#### Data extraction and management

We will extract data independently in duplicate. Two review authors will extract data on the population (including PROGRESS‐Plus characteristics, where applicable), study design, intervention, comparison, outcomes, context/setting, and implementation such as adherence and exposure to the social media‐based interventions and delivery of the intervention. We will resolve disagreements on data extraction by discussion or with a third member of the research team (VW) when necessary.

In order to document and characterise how these interventions aim to change behaviour, we will use the validated behaviour change techniques taxonomy (BCTTv1), developed by Michie 2013. The BCTTv1 is a comprehensive hierarchy of 93 behaviour change techniques (BCTs). Each BCT is defined as an “observable, replicable, and irreducible component of an intervention” intended to modify an individual's behaviour (Michie 2013). BCTs are strategies proposed to encourage the adoption of healthful behaviours (Tate 2015; Myneni 2016). One BCT may target one or more theoretical constructs as a means for explaining how interventions lead to behaviour change (Crane 2015; Myneni 2016; Stacey 2016). Furthermore, BCTs can be used to characterise the training of other populations, including professionals and lay people acting as peer mentors using face‐to face and virtual settings (French 2015; Presseau 2015; Edwards 2016). (See Appendix 2 for more information on BCTs). We will code the BCTs in intervention content descriptions using the behaviour change techniques taxonomy version 1 (BCTTv1) (Michie 2013). We will code BCTs independently using two coders who will receive online BCT training (www.bct‐taxonomy.com). Trainees will need to score at least 70% on the first assessment (after completing the first four sessions of the training) to progress to the next sessions, which we will also use as a standard for demonstrating acceptable understanding of BCTs. We will also have trainees code a sample of studies and use an inter‐rater reliability score of kappa value 0.60 or above to demonstrate the ability to code BCTs with an acceptable level of competence (Landis 1977). We will develop a modified coding manual with coding rules and examples for the BCTs that are relevant and specific to social media‐based interventions. We will group BCTs by themes describing intervention components (e.g. training, social media activities, non‐social media activities).

We will code BCTs separately for each arm of the study (including control), and for the social media components as well as non‐social media components. For example, a social media intervention may be delivered alongside a mass media campaign, which may use different BCTs. We will classify each BCT as present or absent, and we will collect the text to support the judgment for each study. We will contact authors for additional information if the published report and protocols are insufficient to make judgments about BCTs used. We will also assess whether the web platform is still available online (in the version used for the study), and will use this to collect information about the BCTs and social media components.

We will assess the context of the intervention using the Context and Implementation of Complex Interventions (CICI) framework, which captures data on the location, geography, epidemiology, socioeconomic, sociocultural, political, legal, and ethical domains at different levels (from local community to national and international) (Pfadenhauer 2016).

#### Risk of bias

Two independent review authors will assess risk of bias using the Cochrane ‘Risk of Bias’ tool for randomised trials, to collect details on how the study was designed and judge low, unclear, or high risk of bias for each domain using the guidance in the *Cochrane Handbook for Systematic Reviews of Interventions* (Higgins 2011). The domains include: allocation concealment, generation of sequence, blinding of participants, personnel, and outcome assessment, incomplete outcome data, selective reporting, and baseline imbalance. We will also assess protection against contamination since social media crosses geographic borders.

For interrupted time series and controlled before‐and‐after studies, we will use the modified EPOC risk of bias tool (EPOC 2017b), which assesses protection against contamination, recruitment bias, and whether there are unit of analysis errors in studies with allocation by clusters instead of individuals.

### Synthesis procedures and statistical analysis

We will conduct meta‐analysis if it is clinically sensible based on the similarity of interventions, populations, outcomes, and comparators. Disadvantaged populations experience different challenges in using and participating in social media interventions, thus we will consider carefully whether it is suitable to combine different populations.

We will analyse continuous outcomes as mean differences in change from baseline, where possible. If baseline and end of study data are available, we will calculate the change from baseline and associated standard deviation, using the methods in the *Cochrane Handbook for Systematic Reviews of Interventions*. We will analyse dichotomous outcomes, such as participation, as risk ratios.

#### Unit of analysis issues

We will analyse studies at the level of allocation. For cluster‐randomised trials where groups of people are allocated to interventions, we will assess these studies for unit of analysis errors. If there are unit of analysis errors (i.e. analysis at the level of the individual, without adjusting for clustering), we will inflate the standard deviation using the variance inflation factor for each intervention arm, as described in the *Cochrane Handbook for Systematic Reviews of Interventions*, using an intra‐cluster correlation coefficient (ICC) from a similar trial or from a database of ICCs. For dichotomous outcomes, we will use the methods in the *Cochrane Handbook for Systematic Reviews of Interventions* to adjust the numerator and denominator for unit of analysis errors.

#### Dealing with missing data

If data are missing, we will contact authors (e.g. to request standard deviations or numbers of participants, if not provided). We will not impute standard deviations from other studies.

We will document how the included studies handled missing data from participants in our data extraction form. We will not impute values for missing participants. If standard deviations are not reported, we will calculate these using other methods such as the confidence interval and exact P values using the formulae in the *Cochrane Handbook for Systematic Reviews of Interventions*. For studies that meet the eligibility criteria and where we do not have sufficient information for meta‐analyses, we will summarise the results narratively.

For dichotomous outcomes, we will analyse using intention‐to‐treat, thus we will use the full number of randomised individuals as the denominator, assuming that the event did not occur in people who are missing. We will conduct a sensitivity analysis assuming the opposite (i.e. that all missing participants had the event of interest).

For continuous outcomes, we will also apply the intention‐to‐treat method, using the number of participants randomised in the analysis, even if some participants are missing.

#### Assessment of heterogeneity

We will assess heterogeneity with the I^2^ statistic and also visual inspection of the forest plots. We will judge clinical heterogeneity as described below. We will explore heterogeneity using pre‐planned subgroup and sensitivity analyses, as described below.

#### Assessment of reporting biases

For analyses with more than 10 studies, we will construct funnel plots to assess the risk of publication bias.

#### Data synthesis

We will conduct meta‐analyses using Review Manager software (version 5.3) (RevMan 2014). We expect substantial heterogeneity in intervention effects, therefore we will use random‐effects models. We will analyse individually randomised trials and cluster‐randomised trials in the same analyses, taking into account unit of analysis issues as above.

We will conduct separate analyses for controlled before‐and‐after studies and interrupted time series studies.

We expect that social media interventions with different health aims (e.g. diet, physical activity, smoking) will measure different types of outcomes, within the classification of physical health, psychological health, behaviour change, well‐being, mortality, and adverse effects. Within each outcome category, we will assess heterogeneity across the types of intervention, populations, and outcomes to judge whether it is sensible to pool across studies. As above, we expect that social media interventions act on different types of outcomes through the same mechanisms, so it may be reasonable to combine different types of outcomes (e.g. measures of change in different behaviours such as exercise, diet, and smoking). We plan to pool behaviour change outcomes, if possible based on content and statistical judgement, since they represent the same underlying concept of behaviour change and we expect the underlying mechanism of action to be the same. To do this, we will use effect sizes calculated using the standardised mean difference in RevMan 5.3 (Hedges (adjusted) *g*). We plan to pool psychological outcomes since we expect that the underlying concept of psychological stress will be similar, and can thus be combined using SMDs. If our clinical and statistical experts judge that pooling is not reasonable, we will use a narrative synthesis approach.

Since we expect that different scales and tools will be used to measure continuous outcomes, we will analyse continuous outcomes as standardised mean differences, using change from baseline as the measure of effect.

We will document decisions about the classification of outcomes, methods of measurement and selection from amongst multiple measures of the same concept. For example, if exercise behaviour is measured with intensity, frequency, and type of activity (e.g. walking, running, biking), we will choose the outcome measurement that is either: described as primary outcome in the study, used for sample size calculation, or reported more prominently in the abstract, results, or discussion.

We will analyse dichotomous outcomes as risk ratios, using intention‐to‐treat. As above, there may be heterogeneity in the types of outcomes within each outcome category and we will document this.

For studies with multiple arms, we will select the intervention arm that is considered to have the highest intensity of social media interaction (e.g. most frequent interaction or most frequent reminders). Similarly, for the control arm, we will select the arm that has the least exposure to social media. For subgroup analysis of intensity of social media, these studies may be included in the same analysis, and we will divide the participants evenly for the shared intervention arm, as described in the *Cochrane Handbook for Systematic Reviews of Interventions* (Higgins 2011).

We will also assess whether we have sufficient data to construct harvest plots to assess the presence of gradients in effects across sex, ethnicity, socioeconomic status, and other PROGRESS‐Plus characteristics for each outcome (Ogilvie 2008). These plots are helpful in graphically displaying the evidence available for specific populations.

#### Quality of the evidence

We will present the following outcome measures in a ‘Summary of findings’ table: physical health, psychological health outcomes, health behaviours, well‐being, mortality, and any reported adverse effects. We will consult with content experts and a ‘Summary of findings’ table specialist to develop these tables.

We will assess the quality of the body of evidence for each of the outcomes using the GRADE methodology (Guyatt 2011). Using GRADE, we will reflect the extent to which we have confidence, or our level of certainty, that the estimates of effect are correct. We will present our level of certainty as either high, moderate, low, or very low. We will assess the results for each outcome measure against eight criteria. The five criteria considered for possible downgrading of the quality of documentation are: study quality (risk of bias), consistency (consistency between studies), directness (the same study participants, intervention, and outcome measures in included studies are the people, measures, and outcomes we wanted to study), precision of results, and reporting biases. Three criteria may upgrade the level of certainty: strong or very strong associations between intervention and outcome; large or very large dose‐response effects; and where all plausible confounders would have reduced the effect.

As above, we expect that it may not be possible to pool the results. If this is the case, we plan to use the GRADE approach to rate the certainty of evidence using a narrative summary of the effect (Murad 2017).

#### Subgroup analysis and investigation of heterogeneity

As above, we will assess clinical heterogeneity to decide whether statistical meta‐analysis is appropriate. For meta‐analyses, we will assess heterogeneity using visual inspection of forest plots and the I^2^ statistic for heterogeneity.

If sufficient studies are available, we will conduct subgroup analyses for physical health, health behaviours, well‐being, and adverse effects across the following:

- Type of population (healthy, at‐risk, or with a health condition) since having a health condition may provide additional incentive for behaviour change.
- Specific equity characteristics (sex/gender, ethnicity, socioeconomic status, and age are considered the most important for this question).
- Behaviour change techniques (BCTs) used (i.e. we will assess each BCT used in at least two studies as a potential moderator of the effect. This is expected to be fewer than 10 BCTs).
- Presence of mass media concomitant interventions such as campaigns that may magnify the impact of social media interventions if combined.
- Participants (e.g. smokers, under‐users of health services, at‐risk populations, patients with chronic diseases).
- Intensity of social media intervention (e.g. high versus low frequency of interaction, or automatic reminder messages versus no reminders).

We will use the test for subgroup interaction in Review Manager 5.3 to perform these analyses.

We will also assess whether any of these subgroup analyses have been conducted within studies (e.g. to assess effects across sex/gender or socioeconomic status), and report these analyses.

#### Sensitivity analysis

We will conduct sensitivity analysis across risk of bias (i.e. allocation concealment, generation of sequence, and protection against contamination) and for methodological imputations (e.g. adjustment for unit of analysis errors, change score calculations, missing data assumptions).

We will also conduct sensitivity analyses to understand the influence of adherence and participation to social media.

## Acknowledgements

This title is co‐registered with the Cochrane Public Health Group. The protocol authors are grateful for the assistance of Christian Charbonneau (CC) as well as the input of Cochrane Public Health. We also acknowledge the contribution of Jennifer Vincent in the early stages of protocol development.

## SOURCES OF SUPPORT

VW received a peer reviewed Canadian Institutes of Health Research grant as principal investigator for this work. The funder had no role in the design of this review.

## DECLARATIONS OF INTEREST

Vivian Welch ‐ No known conflict of interest.

Jennifer Petkovic ‐ No known conflict of interest.

Rosiane Simeon ‐ No known conflict of interest.

Justin Presseau ‐ No known conflict of interest.

Diane Gagnon ‐ No known conflict of interest.

Alomgir Hossain ‐ No known conflict of interest.

Jordi Pardo Pardo ‐ No known conflict of interest.

Kevin Pottie ‐ No known conflict of interest.

Jennifer Vincent ‐ No known conflict of interest.

Tamara Rader ‐ No known conflict of interest.

Alexandra Sokolovski ‐ No known conflict of interest.

Manosila Yoganathan ‐ No known conflict of interest.

Peter Tugwell ‐ No known conflict of interest.

Marie DesMeules ‐ No known conflict of interest.

## REVIEW AUTHORS

Vivian Welch^1^, Jennifer Petkovic^2^, Rosiane Simeon^2^, Justin Presseau^3^, Diane Gagnon^4^, Alomgir Hossain^5^, Jordi Pardo Pardo^6^, Kevin Pottie^7^, Tamara Rader^8^, Alexandra Sokolovski^9^, Manosila Yoganathan^2^, Peter Tugwell^10^, Marie DesMeules^11^

## ROLES AND RESPONSIBLIITIES

Vivian Welch (VW), Rosiane Simeon (RS), Alexandra Sokolovski (AS), Tamara Rader (TR), Jordi Pardo Pardo (JPP) Manosila Yoganathan (MY), and Jennifer Petkovic (JPetkovic) drafted the protocol. TR developed the search strategy with the assistance of JPP and MY. Input on the protocol draft was provided by the advisory group, including Marie Des Meules (MDM), Diane Gagnon (DG), Lisa Hartling (LH), Heather Manson (HM), Janet Hatcher Roberts (JHR), Alomgir Hossain (AH), Justin Presseau (JPresseau), and Peter Tugwell (PT).

Study selection: MY, AS, RS, VW, CC, J Petkovic

Extracting data from studies: MY, AS, RS, VW, J Petkovic

Entering data in RevMan: MY, CC, VW, J Petkovic

Carrying out analysis: VW, MY, AH

Interpreting analysis: VW, MY, JPP, RS, AS + advisory group

Drafting final review: VW, RS

Disagreement resolution: VW

Updating review: VW

Review advisory group: Rachel Rodin, Robert Geneau

## PRELIMINARY TIMEFRAME

Training and pilot testing on the inclusion criteria: Completion by March 29

Searches for eligible studies: Completion by March 29
Screening the results from the literature search: Completion by May 5
Training and pilot testing the study coding procedure: Completion by May 5
Extraction of data from eligible research reports: Completion by July 15
Statistical analysis: completion by August 15
Preparation of the final review report: completion by September 15

## Plans for updating the review

The review will be updated every two years. VW will be responsible for leading the updates.

## Authors' responsibilities

By completing this form, you accept responsibility for preparing, maintaining and updating the review in accordance with Campbell Collaboration policy. The Campbell Collaboration will provide as much support as possible to assist with the preparation of the review.

A draft review must be submitted to the relevant Coordinating Group within two years of protocol publication. If drafts are not submitted before the agreed deadlines, or if we are unable to contact you for an extended period, the relevant Coordinating Group has the right to de‐register the title or transfer the title to alternative authors. The Coordinating Group also has the right to de‐register or transfer the title if it does not meet the standards of the Coordinating Group and/or the Campbell Collaboration.

You accept responsibility for maintaining the review in light of new evidence, comments and criticisms, and other developments, and updating the review at least once every five years, or, if requested, transferring responsibility for maintaining the review to others as agreed with the Coordinating Group.

## Publication in the Campbell Library

The support of the Campbell Collaboration and the relevant Coordinating Group in preparing your review is conditional upon your agreement to publish the protocol, finished review and subsequent updates in the Campbell Library. Concurrent publication in other journals is encouraged. However, a Campbell systematic review should be published either before, or at the same time as, its publication in other journals. Authors should not publish Campbell reviews in journals before they are ready for publication in the Campbell Library. Authors should remember to include a statement mentioning the published Campbell review in any non‐Campbell publications of the review.

I understand the commitment required to undertake a Campbell review, and agree to publish in the Campbell Library. Signed on behalf of the authors:

Form completed by: Vivian Welch

Date: 23 March 2017

## Footnotes

1 Methods Centre, Bruyère Research Institute, Ottawa, Canada

2 Bruyère Research Institute, University of Ottawa, Ottawa, Canada

3 Clinical Epidemiology Program, Ottawa Hospital Research Institute, Ottawa, Canada

4 Department of Communication, University of Ottawa, Ottawa, Canada

5 Cardiovascular Research Methods Centre, University of Ottawa Heart Institute, Ottawa, Canada

6 Centre for Practice‐Changing Research, Ottawa Hospital Research Institute, The Ottawa Hospital ‐ General Campus, Ottawa, Canada

7 Family Medicine, University of Ottawa, Ottawa, Canada

8 Canadian Agency for Drugs and Technologies in Health (CADTH), Ottawa, Canada

9 Bruyère Research Institute, Ottawa, Canada

10 Department of Medicine, Faculty of Medicine, University of Ottawa, Ottawa, Canada

11 Social Determinants and Science Integration/ Direction des déterminants sociaux et de l'intégration scientifique, Public Health Agency of Canada/Agence de santé publique du Canada, Ottawa, Canada

## 1. Sample search strategy

**Database: Ovid MEDLINE(R) Epub Ahead of Print, In‐Process & Other Non‐Indexed Citations, Ovid MEDLINE(R) Daily and Ovid MEDLINE(R) <1946 to Present> Search Strategy:**

1 exp Social Media/

2 Blogging*.mp. [mp=title, abstract, original title, name of substance word, subject heading word, keyword heading word, protocol supplementary concept word, rare disease supplementary concept word, unique identifier, synonyms]

3 Blogging/

4 Communications Media/

5 Social Networking/

6 (social adj2 media).tw.

7 ((virtual or online) adj2 (communit$ or network$)).tw.

8 “Web 2.0”.tw.

9 Facebook.tw.

10 Twitter.tw.

11 MySpace.tw.

12 Tumblr.tw.

13 instagram.tw.

14 pinterest.tw.

15 wiki$.tw.

16 YouTube.tw.

17 vimeo.tw.

18 Flickr.tw.

19 Delicious.tw.

20 blog$.tw.

21 (linkedin or linked in).tw.

22 (sixdegrees or six degrees).tw.

23 weibo.tw.

24 curediva.tw.

25 connectedliving.tw.

26 patientslikeme.tw.

27 wego.tw.

28 caringbridge.tw.

29 crowd sourc$.tw.

30 crowdsourc$.tw.

31 hash tag$.tw.

32 hashtag$.tw.

33 microblog$.tw.

34 push technolog$.tw.

35 facetime$.tw.

36 Friendster.tw.

37 Gchat.tw.

38 g‐chat.tw.

39 google maps.tw.

40 Kik.tw.

41 reddit$.tw.

42 subreddit$.tw.

43 snapchat$.tw.

44 tweet$.tw.

45 wechat$.tw.

46 whatsapp$.tw.

47 MXit.tw.

48 QQ.tw.

49 Qzone.tw.

50 baidu.tw.

51 viber.tw.

52 Vkontakte.tw.

53 Odnoklassniki.tw.

54 Facenama.tw.

55 (YY and (social adj2 network$)).tw.

56 (QQ and (social adj2 network$)).tw.

57 (vine and (social adj2 network$)).tw.

58 (LINE and (social adj2 network$)).tw.

59 or/1‐58

60 limit 59 to yr=“2001‐ Current”

61 (pre‐intervention$ or preintervention$ or pre intervention$ or post‐intervention$ or postintervention$ or post intervention$).ti,ab.

62 demonstration project$.ti,ab.

63 (pre‐post or pre test$ or pretest$ or posttest$ or post test$ or (pre adj5 post)).ti,ab.

64 trial.ti. or ((study adj3 aim$) or our study).ab.

65 (before adj10 (after or during)).ti,ab.

66 (quasi‐experiment$ or quasiexperiment$ or quasi random$ or quasirandom$ or quasi control$ or quasicontrol$ or ((quasi$ or experimental) adj3 (method$ or study or trial or design$))).ti,ab,hw.

67 (time points adj3 (over or multiple or three or four or five or six or seven or eight or nine or ten or eleven or twelve or month$ or hour$ or day$ or more than)).ab.

68 (time series adj2 interrupt$).ti,ab,hw.

69 pilot.ti.

70 Pilot projects/

71 (clinical trial or controlled clinical trial or multicenter study or randomized controlled trial or pragmatic clinical trial).pt.

72 (multicentre or multicenter or multi‐centre or multi‐center).ti.

73 random$.ti,ab. or controlled.ti.

74 (control adj3 (area or cohort$ or compare$ or condition or design or group$ or intervention$ or participant$ or study)).ab.

75 evaluation studies as topic/ or prospective studies/ or retrospective studies/ or non‐randomized controlled trials as topic/ or interrupted time series analysis/ or controlled before‐after studies/

76 (during adj5 period).ti,ab.

77 ((strategy or strategies) adj2 (improv$ or education$)).ti,ab.

78 (rat or rats or cow or cows or chicken$ or horse or horses or mice or mouse or bovine or animal$).ti.

79 exp animals/ not humans.sh.

80 (or/61‐77) not (or/78‐79)

81 60 and 80

## 2. Identifying and coding behaviour change techniques employed in social media interventions and comparators

To promote successful replication of research findings, it is necessary to clearly define key components of interventions. The difficulty in this lies in the complex nature of most interventions, which are commonly made up of multiple interacting elements (Craig 2008). Studies rarely investigate the effectiveness of each component of the intervention separately, but rather assess the efficacy of the multicomponent intervention as a whole.

As others seek to replicate the results achieved in previous studies, they are faced with the task of deciphering which components of these interventions are important and whether it is necessary to include all components of the previous studies as originally presented. Variation between studies makes it particularly difficult to tease out the critical components in the interventions (Tate 2015).

It is therefore important to understand the specific mechanisms of action, or active ingredients, of these multicomponent interventions, and analysing behaviour change techniques is one method of doing so. A behaviour change technique (BCT) is defined as an “observable, replicable, and irreducible component of an intervention” intended to modify an individual's behaviour (Michie 2013). To allow for simplification and standardisation in the identification and coding of BCTs, Michie 2013 developed the Behavioural Change Technique Taxonomy (BCTTv1).

The BCTTv1 is a comprehensive hierarchy of 93 BCTs, which we will use to code which interventions were used to implement behaviour change. (See table below for BCT examples and how we would document them for a sample study).

Based on previous studies, it is likely that only a fraction of the 93 BCTs will be used within each type of health intervention (Presseau 2015; Edwards 2016). For example, a study looking at gamified health apps for smartphones found that predominantly three of the 16 behaviour change categories were used (51 of the 93 BCTs): feedback and monitoring (used by 94% of applications), reward and threat (used by 81% of applications), and goals and planning (used by 81% of applications) (Edwards 2016).

## BCT examples and definitions

**Table:**

**Behaviour Change Grouping**	**Behaviour Change Technique (BCT)**	**Definitions (** **Michie 2013** **)**	**Example from (** **Graham 2011** **)**
Goals and planning	1.1 Goal setting (behaviour)	“Set or agree on a goal defined in terms of the behavior to be achieved.”	“Provides assistance in setting a quit date”
Goals and planning	1.2 Problem solving	“Analyse, or prompt the person to analyse, factors influencing the behavior and generate or select strategies that include overcoming barriers and/or increasing facilitators (includes ‘Relapse Prevention’ and ‘Coping Planning’).”	“Provides problem solving/skills training content”
Regulation	11.1 Pharmacological support	“Provide, or encourage the use of or adherence to, drugs to facilitate behavior change.”	“Provides tailored assistance in using pharmacotherapies approved by the US Food and Drug Administration”
Social support	3.1 Social support (unspecified)	“Advise on, arrange or provide social support (e.g. from friends, relatives, colleagues, buddies or staff) or non‐contingent praise or reward for performance of the behavior.”	“Provides social support within its large online social network”
Feedback and monitoring	2.3 Self‐monitoring of behaviour	“Establish a method for the person to monitor and record their behavior(s) as part of a behavior change strategy.”	“Users are prompted for updates at each login”
